# Stereotactic ablative radiotherapy for ultra-central lung tumors: prioritize target coverage or organs at risk?

**DOI:** 10.1186/s13014-018-1001-6

**Published:** 2018-04-02

**Authors:** Donna H. Murrell, Joanna M. Laba, Abigail Erickson, Barbara Millman, David A. Palma, Alexander V. Louie

**Affiliations:** 1 Department of Physics and Engineering, London Regional Cancer Program, London, ON Canada; 2 Department of Radiation Oncology, London Health Sciences Centre, 790 Commissioners Road East, London, ON Canada

**Keywords:** Normal tissue complication probability, Ultra-central lung tumor, Stereotactic ablative radiotherapy

## Abstract

**Background:**

Lung stereotactic ablative radiotherapy (SABR) is associated with low morbidity, however there is an increased risk of treatment-related toxicity in tumors directly abutting or invading the proximal bronchial tree, termed ‘ultra-central’ tumors. As there is no consensus regarding the optimal radiotherapy treatment regimen for these tumors, we performed a modeling study to evaluate the trade-offs between predicted toxicity and local control for commonly used high-precision dose-fractionation regimens.

**Methods:**

Ten patients with ultra-central lung tumors were identified from our institutional database. New plans were generated for 3 different hypofractionated schemes: 50 Gy in 5 fractions, 60 Gy in 8 fractions and 60 Gy in 15 fractions. For each regimen, one plan was created that prioritized planning target volume (PTV) coverage, potentially at the expense of organ at risk (OAR) tolerance, and a second that compromised PTV coverage to respect OAR dose constraints. Published radiobiological models were employed to evaluate competing treatment plans based on estimates for local control and the likelihood for toxicity to OAR.

**Results:**

The risk of esophageal or pulmonary toxicity was low (< 5%) in all scenarios. When PTV coverage was prioritized, tumor control probabilities were 92.9% for 50 Gy in 5 fractions, 92.4% for 60 Gy in 8 fractions, and 52.0% for 60 Gy in 15 fractions; however the estimated risk of grade ≥ 4 toxicity to the proximal bronchial tree was 68%, 44% and 2% respectively. When dose to OAR was prioritized, the risk of major pulmonary toxicity was reduced to < 1% in all schemes, but this compromise reduced tumor control probability to 60.3% for 50 Gy in 5 fractions, 65.7% for 60 Gy in 8 fractions and 47.8% for 60 Gy in 15 fractions.

**Conclusions:**

The tradeoff between local control and central airway toxicity are considerable in the use of 3 commonly used hypofractionated radiotherapy regimens for ultra-central lung cancer. The results of this planning study predict that the best balance may be achieved with 60 Gy in 8 fractions compromising PTV coverage as required to maintain acceptable doses to OAR. A prospective phase I trial (SUNSET) is planned to further evaluate this challenging clinical scenario.

**Electronic supplementary material:**

The online version of this article (10.1186/s13014-018-1001-6) contains supplementary material, which is available to authorized users.

## Background

Stereotactic ablative radiotherapy (SABR) is an established treatment for medically inoperable patients with early stage non-small cell lung cancer (NSCLC). Morbidity is low when SABR is employed for tumors that are peripherally located, and risk-adapted schemes can be employed when the lesion is near critical mediastinal structures at increased risk for treatment-related toxicity. The magnitude of such risk has been the subject of debate and the optimal SABR dose-fractionation regimen for central tumors is an active area of research [1, 2]. In the landmark Radiation Therapy and Oncology Group (RTOG) 0236 trial that demonstrated the efficacy of SABR for peripheral lung tumors; a 2 cm ‘no-fly zone’ around the proximal bronchial tree was coined as an area of trial exclusion [3]. Subsequently, the RTOG 0813 dose escalation trial was initiated to investigate the safety of 5-fraction regimens of SABR for central lung tumors, assessing the increase of total doses of 50 to 60 Gy [4]. Though the highest dose level in this trial was achieved, there have been published reports of fatal toxicity after treatment with 45–50 Gy in 5 fractions [5–7]. A more fractionated regimen of 60 Gy in 8 for central lung tumors was investigated by the European Organization for Research and Treatment of Cancer (EORTC) in the phase II trial, LungTech; however, efficacy and toxicity data are not yet reported [8, 9]. Notably, tumors directly abutting or invading the proximal bronchial tree – defined herein as ‘ultra-central’ lung tumors – were not specifically addressed in the RTOG 0813 or LungTech trials and represent what is likely to be a higher-risk subgroup.

Ultra-central lung tumors are a relatively uncommon clinical scenario and reports evaluating SABR outcomes in this context are limited. One study found that 30–40 Gy over 5 fractions resulted in 1-year local control rate of 63% of hilar tumors; even with this moderate dose, a SABR-related death due to bronchial fistula was noted [10]. A similar investigation of 35–40 Gy for lung tumors abutting or invading the mainstem bronchus revealed 70% local control at 1 year, but with no reports of treatment-related death and 1 report of grade 4 atelectasis [11]. Likewise, a retrospective study identified 7 NSCLC patients treated with 50 Gy in 4–5 fractions who all achieved local tumor control at 2 years, without any grade 2 or higher toxicities [12]. In contrast to these results, Haseltine et al. estimated a 22% rate of fatal complications attributable to a 45–50 Gy in 5 fraction SABR regimen when tumors abut the proximal bronchial tree [6]. Finally, a large cohort (*n* = 47), ranging from stage IA-IIIA and with or without nodal disease, was reported by Tekatli et al. where the actuarial incidence of treatment-related death was 16.3% at 12 months for patients who received 60 Gy in 12 fractions [13]. Considering these findings, concern remains over the balance between local control and toxicity in treating ultra-central lung tumors and there is no consensus regarding the most appropriate SABR or hypofractionated RT scheme.

In the absence of robust evidence for the ideal dose-fractionation regimen, radiobiological modeling can be a helpful tool to evaluate the risks and benefits of different treatment plans. Herein, we employ published radiobiological models to evaluate the likelihood of toxicity and estimate local control for SABR dose-fractionation regimens that are commonly used in the treatment of ultra-central lung tumors.

## Methods

### Cohort characteristics

**Fig. 1:**
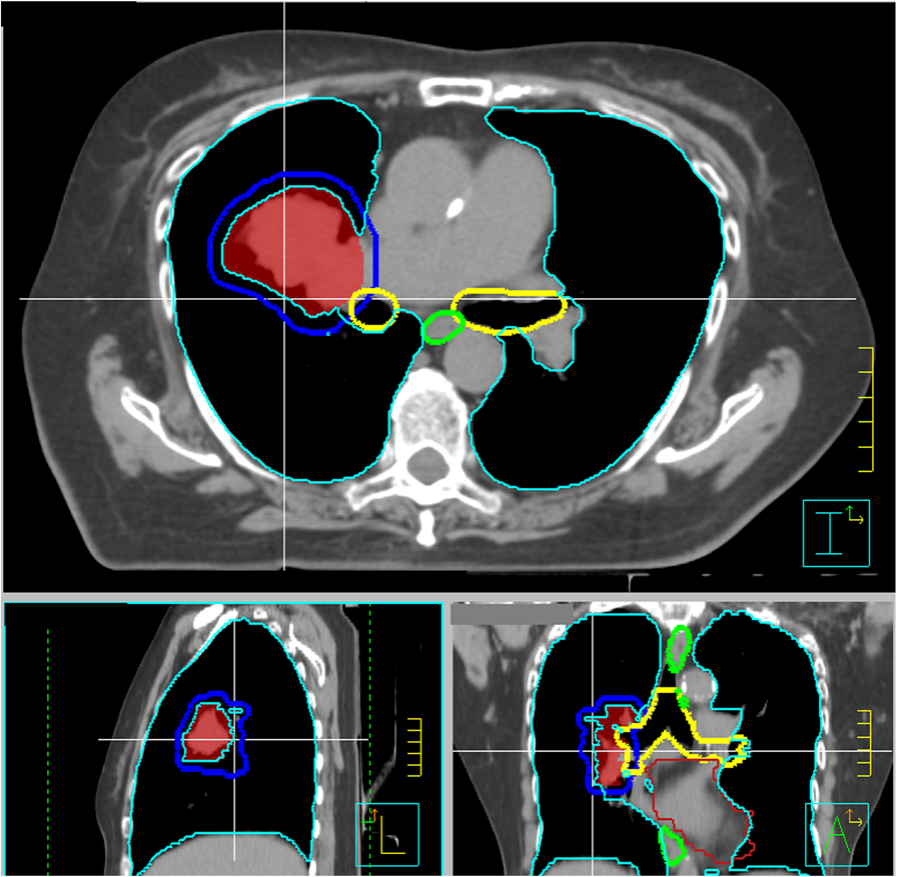
An illustrative example of a contoured ultra-central lung tumor case**.** The relative locations of tumor and OAR are indicated; IGTV (red), PTV (blue), proximal bronchial tree (yellow), esophagus (green), heart (maroon), and healthy lung (cyan). Here, the PTV volume is 83.1 cm^3^ and 2.2 cm^3^ overlap with the proximal bronchial tree

Ten patients with ultra-central lung tumors, defined as tumors with a gross tumor volume (GTV) directly abutting or invading the proximal bronchial tree, treated with RT between November 2013 and November 2016 were identified from our institutional database and included in this research ethics board approved planning study. Clinically delivered doses varied substantially. For two patients, ≥95% of the PTV was covered by 60 Gy in 8 fractions; this dose was prescribed in three additional patients, with compromised PTV coverage. For three patients, ≥98% of the PTV was covered by 95% of 60 Gy in 15 fractions. One patient received 54 Gy in 8 fractions with compromised PTV coverage, and one patient received 40 Gy in 15 fractions. The location of an ultra-central tumor relative to the bronchial tree, and other important organs at risk (OAR), is illustrated in Fig. 1 with axial, sagittal, and coronal computed tomography (CT) slices.

### CT simulation

Patients were immobilized in the supine position, with their arms above their head. Fast helical CT Simulation (CT-SIM) scans were acquired, as well as a 4DCT scan to assess respiratory motion. The untagged average, maximum inspiration, and maximum expiration CT datasets were transferred to the Pinnacle treatment planning system (software version 9.10, Philips Healthcare, Fitchburg, WI, USA). Delineation of target volumes and OARs were completed by a thoracic radiation oncologist (JL). The GTV was contoured on the maximum inspiration and maximum expiration 4DCT images and combined to form the internal gross tumor volume (IGTV) to account for tumor motion. A 5 mm expansion on the IGTV was applied to create the planning target volume (PTV) for treatment planning.

### Treatment planning

Experienced dosimetrists (AE, BM) generated new radiation treatment plans for each of the 10 cases using three commonly used hypofractionated schemes: 50 Gy in 5 fractions, 60 Gy in 8 fractions, and 60 Gy in 15 fractions. An inhomogeneous dose prescription was used to create SABR plans for 50 Gy in 5 fraction and 60 Gy in 8 fraction regimens; whereas a traditional homogeneous prescription was used for 60 Gy in 15 fractions. Two plans were generated for each of the three dose regimens: one prioritizing tumor coverage (with dose to OARs as low as reasonably achievable) and the other plan compromising PTV coverage in order to respect the dose constraints for OARs. OARs included the proximal bronchial tree, esophagus, great vessels, heart, spinal cord and lungs. All plans were created using volumetric modulated arc therapy (VMAT) with two arcs covering 180–360° using 6 or 10 MV photon beams. The dose constraints for OAR were based on Timmerman’s summary for 5-fraction SBRT and were corrected for dose-fractionation using biologically effective dose calculations (Table 1) [14]. Descriptive statistics were generated to illustrate the dosimetric trade-offs of each scenario.

**Table 1:** Dose objectives for each fractionation regimen, based on standard linear-quadratic model biologically effective doses calculated with α/β=3

Structure	Volume (cm^3^)	5 fraction		8 fraction		15 fraction	
		D_V_ (Gy)	D_max_ (Gy)	D_V_ (Gy)	D_max_ (Gy)	D_V_ (Gy)	D_max_ (Gy)
Trachea and ipsilateral bronchus	< 4	18 (3.6/fx)	38 (7.6/fx)	21 (2.6/fx)	46 (5.8/fx)	25.5 (1.7/fx)	58.5 (3.9/fx)
Spinal cord	< 0.25	22.5 (4.5/fx)	30 (6.0/fx)	26.5 (3.3/fx)	36 (4.5/fx)	32 (2.1/fx)	45 (3.0/fx)
Spinal cord	< 1.2	13.5 (2.7/fx)		15.5 (1.9/fx)		18 (1.2/fx)	
Esophagus	< 5	27.5 (5.5/fx)	35 (7.0/fx)	32.5 (4.1/fx)	42 (5.3/fx)	41 (2.7/fx)	53 (3.5/fx)
Ipsilateral brachial plexus	< 3	30 (6.0/fx)	32 (6.4/fx)	36 (4.5/fx)	38.5 (4.8/fx)	45 (3.0/fx)	48 (3.2/fx)
Heart/pericardium	< 15	32 (6.4/fx)	38 (7.6/fx)	38.5 (4.8/fx)	46 (5.8/fx)	48 (3.2/fx)	58 (3.9/fx)
Great vessels	< 10	47 (9.4/fx)	53 (10.6/fx)	57.5 (7.2/fx)	65 (8.1/fx)	74 (4.9/fx)	84 (5.6/fx)
Skin	< 10	30 (6.0/fx)	32 (6.4/fx)	36 (4.5/fx)	38.5 (4.8/fx)	45 (3.0/fx)	48 (3.2/fx)
Lung (right and left)	1500	12.5 (2.5/fx)		14 (1.8/fx)		16.5 (1.1/fx)	
Lung (right and left)	1000	13.5 (2.7//fx)		15.5 (1.9/fx)		18 (1.2/fx)	

*D*_*V*_ allowable dose to the volume, *D*_*max*_ maximum point dose, *fx* fraction

### Radiobiological modeling

Treatment plans were evaluated using published radiobiological models for normal tissue complication probability (NTCP) and tumor control probability (TCP). Differential dose volume data was exported from Pinnacle in 1 cGy bins for modeling. To correct for differences in dose fractionation, physical doses were converted to isoeffective doses in 2 Gy per fraction (also termed the equivalent dose in 2 Gy per fraction; EQD_2_) using α/β=3 for late responding normal tissue and α/β=10 for tumor (Eq. 1). For each fractional volume, D_i_ is the total physical dose and d_i_ is the physical dose per fraction.

1 $$ {EQD}_2={D}_i\frac{1+\frac{d_i}{\raisebox{1ex}{$\alpha $}\!\left/ \!\raisebox{-1ex}{$\beta $}\right.}}{1+\frac{2}{\raisebox{1ex}{$\alpha $}\!\left/ \!\raisebox{-1ex}{$\beta $}\right.}} $$

Lyman-Kutcher-Burman models were used to estimate the risk for acute esophageal toxicity greater than grade 2 by RTOG scoring, symptomatic pneumonitis of all grades, and pericarditis or pericardial effusion, using the Mohan formulation (Eqs. 2, 3 and 4) [15].

2 $$ NTCP=\frac{1}{\sqrt{2\pi }}\kern0.5em {\int}_{-\infty}^t{e}^{\frac{-{x}^2}{2}}\  dx $$

3 $$ t=\frac{D_{eff}-{TD}_{50}}{m{TD}_{50}} $$

4 $$ {D}_{eff}={\left(\sum \limits_i{v}_i{D_i}^{\raisebox{1ex}{$1$}\!\left/ \!\raisebox{-1ex}{$n$}\right.}\right)}^n $$

In this model, TD_50_ is the uniform dose to an organ associated with 50% complication risk, m describes slope of the dose-response, n describes volume effect, and D_i_ is the dose (EQD_2_) to fractional volume, v_i_. NTCP were estimated using published parameters by Belderbos et al. for esophagitis (TD_50_ = 47 Gy, *n* = 0.69, m = 0.36), Semenko et al. for pneumonitis (TD_50_ = 29.9 Gy, *n* = 1, m = 0.41) and Martel et al. for pericarditis (TD_50_ = 50.6 Gy, *n* = 0.64, m = 0.13) [16–18]. Additional model parameters published by Chapet, Zehentmayr, Seppenwoolde, and Burman were also evaluated for comparison (Additional file 1: Table S1) [19–22].

To our knowledge, there are no published model parameters for determining risk for proximal bronchial tree toxicity. We present risk estimates based on average minimum dose (EQD_2_) to 1cm^3^, 2cm^3^, and 3cm^3^ of the proximal bronchial tree for each treatment regimen compared against published probability curves for grade 4 or 5 proximal bronchial tree toxicity at two years, which included outcomes such as fatal hemoptysis [23].

TCP was calculated using the Martel model (Eq. 5) which describes the dose-response curve for NSCLC and estimates progression-free survival at 30 months [24].

5 $$ TCP\ (D)=\frac{1}{1+{\left(\raisebox{1ex}{${D}_{50}$}\!\left/ \!\raisebox{-1ex}{${D}_i$}\right.\right)}^{4\gamma }} $$

In this model, D_i_ is the uniform dose irradiated to a fractional volume, D_50_ is the dose needed to achieve a 50% probability of tumor control, and γ is the normalized slope of the sigmoid-shaped dose response curve at D_50_. Input parameters published by Fowler et al. (D_50_ = 70 Gy, γ=1.94) were used to improve applicability of the Martel model to SABR regimens [25].

The probability for uncomplicated tumor control (UTCP) was calculated, as previously described, to quantify the most desirable treatment regimen (Eq. 6) [26].

6 $$ UTCP= TCP\ast \left(1- NTCP\right) $$

## Results

Relevant doses to the proximal bronchial tree for each treatment regimen are reported in Table 2. When dose to the organs at risk was prioritized, PTV coverage by the 100% isodose line for the 50 Gy in 5 fraction regimen was reduced from 96.0% (95.0–100) to 67.8% (19.6–89.2); for 60 Gy in 8, from 96.0% (95.0–99.9) to 68.5% (18.8–89.5); and for 60 Gy in 15 fractions, coverage by the 95% isodose line was reduced from 97.3% (95.5–100) to 91.6% (82.0–97.8). One patient with a large (85.8 cm^3^ PTV) ultra-central tumor located close to the spinal cord required significant compromise of tumor coverage in order to respect the dose constraints for organs at risk. The range of PTV coverage for the other nine patients ranged from 56.8–89.2% for 50 Gy in 5, 59.4–89.5% for 60 Gy in 8 and 82.0–97.8% for 60 Gy in 15 fractions.

**Table 2:** Doses received by the proximal bronchial tree across competing treatment plans

parameter	PTV coverage prioritized			OAR constraints prioritized		
	50 Gy in 5	60 Gy in 8	60 Gy in 15	50 Gy in 5	60 Gy in 8	60 Gy in 15
D_max_ (Gy)	60.1 (52.3–72.9)	70.1 (63.8–78.1)	60.8 (57.0–62.8)	36.9 (31.5–38.0)	44.7 (38.4–48.2)	57.6 (56.2–58.4)
D_2%_ (Gy)	50.0 (43.9–57.0)	58.7 (48.6–68.8)	55.8 (47.4–61.3)	26.8 (20.8–31.0)	35.0 (25.0–54.6)	47.9 (29.9–56.9)
D_0.1cc_ (Gy)	56.7 (51.1–66.7)	66.4 (57.4–75.2)	59.6 (56.4–62.0)	32.0 (26.8–34.7)	38.9 (32.2–42.0)	55.8 (51.2–57.5)
D_1cc_ (Gy)	47.2 (41.5–55.0)	55.2 (44.7–66.0)	53.1 (44.2–60.9)	25.4 (19.6–30.6)	30.9 (23.5–36.9)	47.4 (38.5–56.4)
D_2cc_ (Gy)	38.1 (27.7–49.8)	44.2 (27.7–59.8)	43.9 (30.1–59.9)	20.3 (16.4–25.2)	24.9 (19.4–30.4)	36.0 (29.3–45.3)
D_3cc_ (Gy)	31.3 (8.3–45.7)	36.5 (8.3–54.9)	34.9 (17.1–58.6)	16.5 (6.4–20.1)	20.3 (7.6–24.3)	25.8 (13.0–29.7)

Cells are presented as the average (range) values

**Fig. 2:**
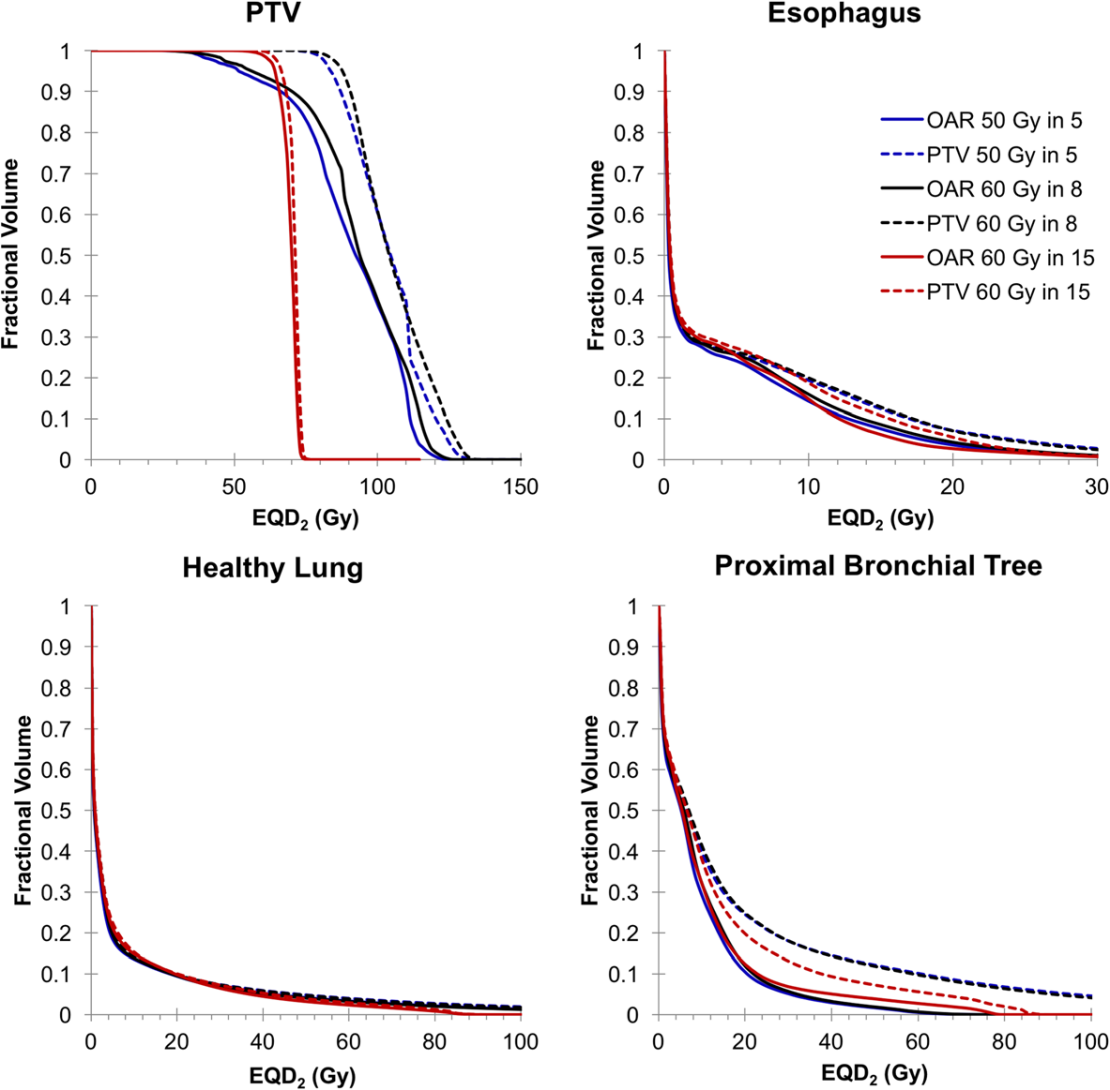
Dose-volume relationships in 2 Gy per fraction for competing dose-fractionation regimens, based on α/β =3 for OAR and α/β = 10 for tumor. Data are graphed at 50 cGy resolution and are expressed as the median (PTV) or average (OAR) fractional volumes to illustrate the relative differences in PTV coverage and OAR sparing between the treatment plans

Cumulative dose-volume relationships for PTV, esophagus, healthy lung, and proximal bronchial tree are shown in Fig. 2 for the six competing treatment plans. SABR, when delivered as 50 Gy in 5 fractions or 60 Gy in 8 fractions, required substantial compromise of PTV coverage to satisfy tolerance doses to OAR. A comparison of the OAR-prioritized with PTV-prioritized plans illustrates an appreciable difference in dose to the proximal bronchial tree and esophagus for all fractionation regimens, whereas dose to the lung was similar regardless of plan or prioritization.

A simple evaluation of EQD_2_ (based on α/β = 10) for each fractionation schedule, if homogeneous dose is assumed, results in 87.5 Gy, 83.3 Gy, and 70 Gy to tumor for 60 Gy in 8 fractions, 50 Gy in 5 fractions, and 60 Gy in 15 fractions, respectively. The derived TCP values are 85, 80, and 50, suggesting TCP is most favorable for 60 Gy in 8 fractions.

The NTCP and TCP results are presented in Table 3. The mean probability of acute grade ≥ 2 esophageal toxicity, pneumonitis, and pericarditis or pericardial effusion was less than 5% in all dose-fractionation regimens, regardless of PTV or OAR prioritization. If PTV coverage was not compromised with SABR, the mean estimated risk of grade 4 or 5 toxicity to the proximal bronchial tree based on D_1cc_ was high at 49.7% and 42.7% for 50 Gy in 5 fractions and 60 Gy in 8 fractions, respectively, and exceeded 70% for 4 of 10 patients. The PTV-prioritized 60 Gy in 15 fraction regimen had a risk of 4.0%. When dose to OAR was prioritized, the risk of toxicity to the proximal bronchial tree was reduced to 1% or less for all 3 fractionation schemes. This compromise resulted in a reduction in mean TCP compared to PTV-prioritized plans from 92.9% to 60.3% for 50 Gy in 5 fractions, 92.4% to 65.7% for 60 Gy in 8 fractions, and 52.0% to 47.8% for 60 Gy in 15 fractions.

**Table 3:** Radiobiological modeling results for tumor control probability and normal tissue complication probability across competing dose-fractionation regimens

Outcome	PTV coverage prioritized			OAR constraints prioritized		
	50 Gy in 5	60 Gy in 8	60 Gy in 15	50 Gy in 5	60 Gy in 8	60 Gy in 15
tumor control	92.9 (89.7–99.1)	92.4 (84.7–95.3)	52.0 (48.3–55.7)	60.3 (31.5–80.1)	65.7 (39.2–81.4)	47.8 (41.0–53.3)
acute esophagitis ≥ grade 2	1.18 (0.45–3.98)	1.05 (0.44–2.44)	0.86 (0.42–1.85)	0.72 (0.34–1.35)	0.77 (0.39–1.40)	0.72 (0.42–1.49)
symptomatic pneumonitis (all grades)	4.30 (1.79–11.6)	4.09 (1.90–11.1)	3.68 (1.53–13.8)	3.44 (2.24–8.03)	3.45 (1.75–8.08)	2.97 (1.54–6.84)
pericarditis or pericardial effusion	0.01 (0.00–0.08)	0.00 (0.00–0.00)	0.00 (0.00–0.00)	0.00 (0.00–0.00)	0.00 (0.00–0.00)	0.00 (0.00–0.00)
proximal bronchial tree toxicity (grade 4 or 5)	49.7 (15.0–70.0^a^)	42.7 (5.0–70.0^a^)	4.0 (0.0–10.0)	0.0 (0.0–0.0)	0.0 (0.00–0.0)	1.3 (0.0–5.0)
uncomplicated tumor control	46.7 (27.6–77.6)	53.5 (25.4–90.4)	49.9 (44.6–54.0)	60.3 (31.5–80.1)	65.7 (39.2–81.4)	47.2 (41.0–52.8)

Cells are average (range) tumor control probability or normal tissue complication probability (%). ^a^4 cases had an EQD_2_ greater than the range presented by Cannon et al and it is therefore likely that their risk is greater than 70%

The most favourable UTCP regarding proximal bronchial tree injury was 65.7% with 60 Gy in 8 fractions prioritizing OAR. Notably, the previously mentioned case with substantial compromise of PTV coverage had especially low UTCP for this regimen; when excluded, the favourability of this treatment plan increases with minimum UTCP greater than 44% for the remaining cases and 69% on average. The mean UTCP for the other treatment plans were 46.7%, 53.5%, and 49.9% for 50 Gy in 5 fractions, 60 Gy in 8 fractions, and 60 Gy in 15 fractions, respectively, when PTV coverage was prioritized. When OARs were prioritized, the UTCP increased to 60.3% for 50 Gy in 5, but decreased to 47.2% for 60 Gy in 15 fractions.

**Fig. 3:**
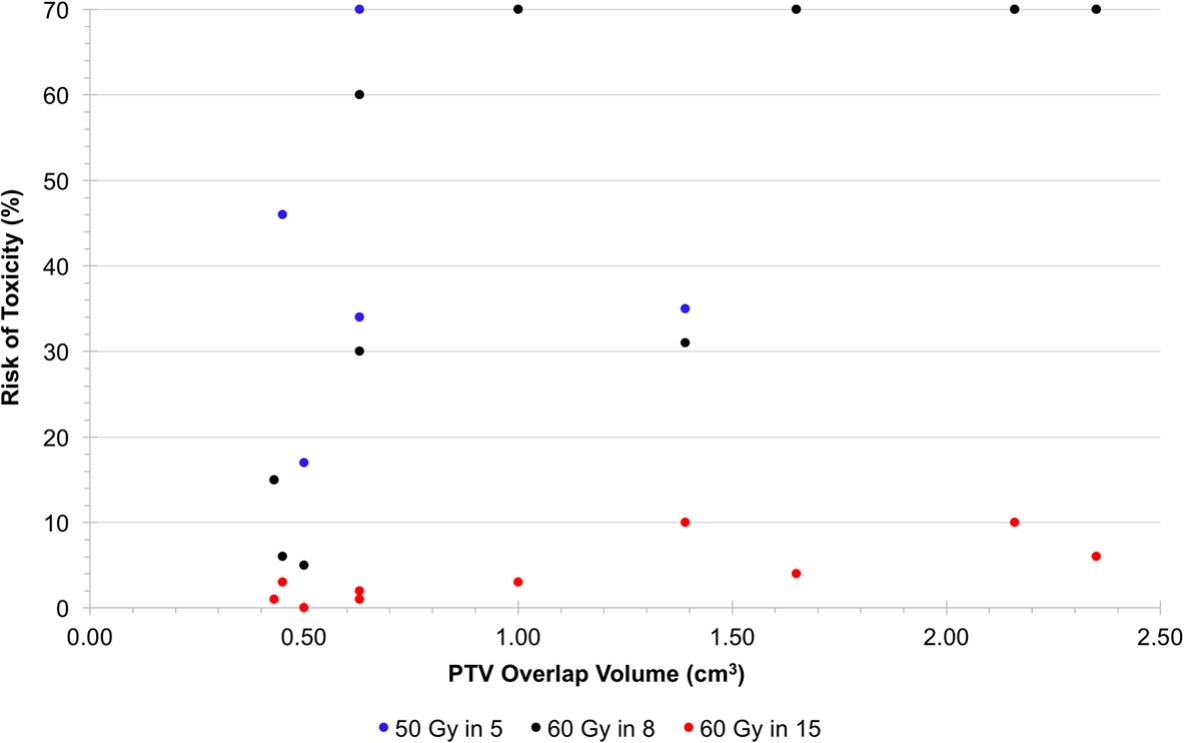
Relationship between likelihood of proximal bronchial tree injury and the tumor overlap volume. The risk of grade 4 or 5 toxicity to the proximal bronchial tree based on D_1cc_ vs. PTV overlap volume with the proximal bronchial tree for plans that prioritize PTV coverage

The mean IGTV and PTV volumes were 30.1 cm^3^ (range 7.1–83.1 cm^3^) and 78.8cm^3^ (25.0–171.5 cm^3^), respectively. The mean volume of IGTV and PTV overlap with the proximal bronchial tree were 0.2 cm^3^ (0.01–0.5 cm^3^) and 1.1 cm^3^ (0.4–2.4 cm^3^), respectively. A comparison of PTV overlap volume with likelihood of proximal bronchial tree toxicity suggests a trend for low risk (< 10%) across all overlap volumes studied (up to 2.5 cm^3^) for 60 Gy in 15 fractions. In contrast, substantially greater variability existed for the other dose-fractionation regimens and risk may exceed 70% when overlap volume is greater than 1.5 cm^3^ (Fig. 3).

## Discussion

Consideration of the competing risks for local tumor failure and acute treatment toxicity is required when using hypofractionated radiotherapy or SABR to treat ultra-central lung tumors. This planning study suggests that SABR for ultra-central tumors is feasible, and a risk-adapted approach of 60 Gy in 8 fractions prioritizing OAR tolerance over PTV coverage may provide an acceptable balance between tumor control and toxicity. Although PTV coverage was compromised in this regimen, TCP was reasonable and risk to the proximal bronchial tree was negligible; however, given that proximal bronchial tree injury is correlated with high doses to small volumes (1-3 cm^3^), it should be emphasized that favorable toxicity heavily relies on good image-guidance to ensure the high-dose gradient adequately spares this structure. Alternatively, if treatment set up is difficult, or tumors are large and multiple OARs are a concern, a conservative approach of 60 Gy in 15 fractions prioritizing PTV coverage is reasonable, acknowledging this provides safe doses to OAR at the cost of low TCP compared to other regimens.,

There are a few important limitations to this work. First, only three dose-fractionation regimens were compared and there are other potential dose fractionation schemes which may be appropriate in this setting; however, the three regimens selected for this study are commonly used in practice. Secondly, the small sample size in this exploratory study precludes rigorous statistical analysis and a larger confirmatory study is needed; still, given the paucity of data for this clinical situation, we believe this study provides useful information for physicians treating ultra-central lung tumors. In addition, while careful consideration was given to select models that were relevant to our cohort and the practice at our institution, interpreting results from model-based predictions should be done cautiously as these estimates are derived from outcome data at other centers and can be sensitive to host factors. The model-based predictions presented here are meant to give an estimate for tumor control and toxicity; further prospective clinical research is needed to better assess the true therapeutic ratio of different dose fractionation schemes for ultra-central lung tumors and inform clinical decision-making.

To prospectively address this clinical issue, the Canadian Pulmonary Radiotherapy Group (CAPRI) has launched the Stereotactic body radiotherapy for ultra-central non-small cell lung cancer: A safety and efficacy trial (SUNSET trial, NCT03306680). SUNSET is a multi-centre phase 1 dose escalation study using the continuous re-assessment method, to determine the maximally tolerated SABR dose associated with 30% or lower rate of grade ≥ 3 toxicity within 2 years. This trial will investigate a dose of 60 Gy starting in 8 fractions and escalate to 5 fractions (with de-escalation down to 15 fractions, if necessary). Our modeling results suggest only 4 of the 10 patients included in our study would meet the SUNSET outcome objectives at level 1 (60 Gy in 8 fractions). Additional data from the EORTC LungTech phase II trial of SABR (60 Gy in 8 fractions) for central early stage lung cancer are also awaited (NCT01795521). Until the efficacy and toxicity analysis from these trials are complete, the best dose-fractionation regimen for ultra-central tumors remains unclear.

## Conclusions

In balancing the competing concerns over acute toxicity and local control when treating an ultra-central tumor with SABR, we propose a regimen of 60 Gy in 8 fractions, compromising PTV coverage to respect OAR dose. Alternatively, 60 Gy in 15 fractions represents a conservative and safe approach with the caveat of lower tumor control. Further prospective research is needed to determine the true dose-response relationships associated with ultra-central tumors and elucidate the optimal dose-fractionation regimen.

## Additional file

Additional file 1: **Table S1**. Radiobiological modeling parameters and results for normal tissue complication probability across competing dose-fractionation regimens. (DOCX 20 kb)

## Abbreviations

4DCT: 4-dimensional computed tomography
CT: Computed tomography
CT-SIM: Computed tomography simulation
EORTC: European organization for research and treatment of cancer
GTV: Gross tumor volume
Gy: Gray
IGTV: Internal gross target volume
NTCP: Normal tissue complication probability
PTV: Planning target volume
RTOG: Radiation therapy and oncology group
SABR: Stereotactic ablative radiotherapy
TCP: Tumor control probability
VMAT: Volumetric modulated arc therapy
