# Supplementary material for: Stereotactic ablative radiotherapy for ultra-central lung tumors: prioritize target coverage or organs at risk?
Source: Radiat Oncol. 2018 Apr 2;13:57. doi: 10.1186/s13014-018-1001-6 (PMC5880025; doi:10.1186/s13014-018-1001-6)
Supplement: Supplementary file 1 — Table S1. Radiobiological modeling parameters and results for normal tissue complication probability across competing dose-fractionation regimens. (DOCX 20 kb) [file 13014_2018_1001_MOESM1_ESM.docx]

**Additional File 1**

**Table S1:** Radiobiological modeling parameters and results for normal tissue complication probability across competing dose-fractionation regimens.

| **Toxicity** | **TD_50_** | **n** | **m** | **PTV coverage prioritized** | | | **OAR constraints prioritized** | | |
| --- | --- | --- | --- | --- | --- | --- | --- | --- | --- |
|  |  |  |  | **50 Gy in 5** | **60 Gy in 8** | **60 Gy in 15** | **50 Gy in 5** | **60 Gy in 8** | **60 Gy in 15** |
| acute esophagitis ≥ grade 2^A^ | 51 | 0.44 | 0.32 | 1.15  (0.20-60.8) | 0.83  (0.20-2.59) | 0.55  (0.19-1.35) | 0.46  (0.13-1.14) | 0.50  (0.17-1.18) | 0.43  (0.19-1.18) |
| acute esophagitis ≥ grade 2^B^ | 44.9 | 0.34 | 0.34 | 3.26  (0.43-18.9) | 2.23  (0.45-7.89) | 1.36  (0.42-3.62) | 1.15  (0.28-3.20) | 1.27  (0.36-3.26) | 1.06  (0.40-3.07) |
| pneumonitis^C^ | 30.8 | 1 | 0.37 | 2.80  (0.97-8.61) | 2.63  (1.04-8.15) | 2.39  (0.81-10.5) | 2.13  (1.26-5.61) | 2.14  (0.95-5.66) | 1.79  (0.81-4.65) |
| pericarditis^D^ | 48 | 0.35 | 0.10 | 1.25  (0.00-12.5) | 0.00  (0.00-0.02) | 0.00  (0.00-0.00) | 0.00  (0.00-0.00) | 0.00  (0.00-0.00) | 0.00  (0.00-0.00) |
| proximal bronchial tree (D_2cc_)^E^ |  |  |  | 22.1  (0.0-78.0^†^) | 20.5  (0.0-78.0^‡^) | 1.9  (0.0-10.0) | 0.0* | 0.0* | 0.0  (0.0-0.0) |
| proximal bronchial tree (D_3cc_)^E^ |  |  |  | 12.9  (0.0-66.0) | 11.1  (0.0-58.0) | 1.1  (0.0-9.0) | 0.0* | 0.0* | 0.0* |

Cells are average (range) tumor control probability or normal tissue complication probability (%). *All cases had an EQD_2_ less than the range presented by Cannon et al.; ^†^2 cases and ^‡^1 case had EQD_2_ greater than the range presented by Cannon et al. and it is therefore likely that the risk is greater than 78%.

^A^Chapet et al.; ^B^Zehentmayr et al.; ^C^Seppenwoolde et al.; ^D^Burman et al.; ^E^Cannon et al.
